# Photoplethysmography-based atrial fibrillation detection in patients after crytpogenic stroke

**DOI:** 10.3389/fstro.2024.1496003

**Published:** 2024-12-05

**Authors:** Marthe J. Huntelaar, Jasper L. Selder, Luuk H. G. A. Hopman, Marieke C. Visser, Cornelis P. Allaart

**Affiliations:** ^1^Department of Cardiology, Amsterdam UMC, location VUmc, Amsterdam, Netherlands; ^2^Department of Neurology, Amsterdam UMC, location VUmc, Amsterdam, Netherlands

**Keywords:** cryptogenic stroke, atrial fibrillation, photoplethysmography (PPG), Holter monitoring, screening for AF

## Abstract

**Introduction:**

Undiagnosed atrial fibrillation (AF) is a potential underlying cause of cryptogenic stroke. Prolonged screening for AF using a photoplethysmography (PPG) smartwatch might offer a solution for detecting AF in patients with cryptogenic stroke. In this study, we aim to investigate this strategy by comparing AF detection rates using a PPG-smartwatch and 48 h Holter monitor.

**Methods:**

From December 2019, patients with cryptogenic stroke were included to undergo 28 days of semi-continuous AF monitoring using a Fitbit smartwatch with a PPG-based FibriCheck algorithm, with simultaneous Holter monitoring during the first 48 h. From April 2021, a detailed screening log was installed to characterize potential study participants.

**Results:**

After logged screening of 1,312 patients, enrollment was prematurely halted due to slower-than-expected inclusion rates. 40.8% of the screened patients had cryptogenic stroke, of which 92.5% were non-eligible for inclusion due to logistical, technological, and study-related challenges. Of the 43 patients enrolled, 37 completed PPG monitoring using a smartwatch. 43% of patients had PPG-detected AF in the 28 days after cryptogenic stroke. During the first 48 h, PPG-based screening detected AF in 2 patients, whereas no AF was detected using concurrent Holter monitoring.

**Conclusion:**

The PPG-smartwatch detected AF in 43% of the participants after cryptogenic stroke. However, discrepancies with concurrent Holter monitoring raise major concerns about the accuracy of the detected PPG-based AF. Moreover, the feasibility of a PPG-based screening strategy is limited due to logistical and technological challenges, partly inherent to cryptogenic stroke patients.

## Introduction

Ischemic stroke is one of the leading causes of death and disability, with 20%−40% of cases remaining unexplained even after comprehensive diagnostic evaluation, resulting in the classification of cryptogenic stroke (Sanna et al., 2014). Atrial fibrillation (AF), a well-recognized cause of ischemic stroke, can significantly impact therapeutic decisions when detected in these patients. However, AF is often intermittent and asymptomatic, rendering it a silent risk factor that easily evades detection. Current ESC guidelines recommend continuous ECG monitoring for at least 72 h in cryptogenic stroke patients to screen for AF (Hindricks et al., 2021). In the latest guidelines for stroke prevention from the American Heart Association and American Stroke Association, 30 days of heart rhythm monitoring for AF detection post stroke is suggested (Kernan et al., 2014).

Heart rhythm monitoring can be performed using photoplethysmography (PPG), commonly integrated in smartwatches. PPG is a non-invasive, optical technique that detects blood volume variations from pulsatile blood flow, providing a signal for heart rate monitoring (Pereira et al., 2020). However, while PPG can suggest the presence of AF through irregular pulse detection, it cannot independently confirm AF, which requires an ECG for definitive diagnosis (Hindricks et al., 2021).

Automated analysis of PPG data is preferred due to the vast amount of data generated. One of the various algorithms available is the FibriCheck algorithm, a deep-learning based system to classify PPG measurements. FibriCheck has been evaluated in various studies, demonstrating high sensitivity and specificity for detection of AF under controlled conditions (Selder et al., 2020, 2023). Despite promising results in clinical studies, the reliability and accuracy of PPG-smartwatch measurements automatically analyzed by algorithms during real-world conditions remain less well-established and warrant further investigation.

The primary aim of this study was to evaluate whether 28 days of monitoring using a PPG-smartwatch resulted in more PPG-based AF detection compared to standard Holter monitoring. The secondary objectives were assessing the correlation between AF detection using a PPG-smartwatch and Holter monitoring, and to evaluate rates of newly developed AF and stroke at 3 year follow-up.

## Methods

### Study design, participants

This prospective observational study included patients of 18 years or older with a diagnosis of cryptogenic stroke. The study was conducted at the Amsterdam UMC. Exclusion criteria were lacunar infarction, significant stenosis of the extra- or intracranial circulation, known left ventricular thrombus, known endocarditis with vegetations, recent myocardial infarction, dissection, or drug abuse. Additionally, patients with a prior diagnosis of AF or *de novo* AF detected during hospital admission were excluded. If none of these diagnoses were identified upon discharge, the stroke was classified as cryptogenic. Patients were approached for participation, and provided there were no logistical barriers, informed consent was obtained.

### Data acquisition and reporting

After discharge from the Amsterdam UMC, participants underwent 28 days of semi-continuous PPG recordings (1 min recordings, every 9 min) using a Fitbit Ionic or Fitbit Versa 2 (Fitbit Inc, San Francisco, USA). The FibriCheck algorithm was installed as a clockface on the Fitbit, allowing semi-continuous measurements and the use of additional software (Selder et al., 2023). The initial 48 h of PPG recordings were conducted concurrently with 48 h Holter monitoring for comparison (Fysiologic, ACE Health AB, Johanneshov, Sweden). Holter data were analyzed by trained Holter analysists from Fysiologic. For each patient, PPG recordings were initially analyzed using the most recent version of the FibriCheck algorithm at the time of data acquisition (ranging from v1.2.1 to v1.5.2, Qompium, Hasselt, Belgium). After termination of inclusion, all data for each patient were re-analyzed using FibriCheck v1.5.2. These are the PPG data presented in the results section.

The FibriCheck algorithm assesses PPG recordings and classifies into (1) regular sinus rhythm (SR), (2) non-AF arrhythmias, (3) possible AF in case of sufficient quality, or (4) insufficient quality. If a non-sinus rhythm episode of sufficient quality is detected, the recording was also evaluated by a trained FibriCheck technician. A more extensive description of the algorithm was provided by Selder and colleagues (Selder et al., 2020). As pre-specified in the protocol, no clinical action was undertaken based on the PPG data, and the results of the PPG analysis were not communicated to the participants. Three years after enrollment, a review of clinical data will be undertaken to determine the incidence of newly detected AF and stroke among participants during the follow-up period.

### Data analysis

Power calculation was performed based on the study by Gladstone et al. (2014). Using a two-sided McNemar's Z-test, a sample size of 90 patients achieves 80% power with a type 1 error of 5% and an estimated 15% unusable data. PPG findings were exported from the FibriCheck platform and analyzed and visualized using MatLab (The MathWorks Inc, Natick, Massachusetts, United States). Changes in diagnosis between different versions of the algorithms were determined using Matlab. AF burden was calculated as the number of episodes classified as AF divided by the total number of episodes measured. Baseline characteristics were analyzed using SPSS version 28.0 (IBM SPSS Statistics, Armonk, New York, United States).

Normally distributed data were reported as mean (± standard deviation), non-normally distributed data were reported as median [IQR]. Categorical data were expressed as frequency (percentage).

## Results

### Patient population

Patient enrollment started in December 2019. After 16 inclusions in 16 months, a detailed screening log was implemented to document reasons for non-eligibility and non-participation, starting April 2021. Between December 2019 and January 2024, 43 patients were enrolled in the study, of whom 37 completed PPG-smartwatch measurements (baseline characteristics are presented in Table 1).

**Table 1:** Baseline characteristics.

**Baseline characteristics (*****N*** = **37)**	
**Male (%)**	19 (51.4%)
**Age**	64.2 ± 10.5
**BMI**	26.4 ± 4.0
**CHADSVASC score**	
1	6 (16.2%)
2	14 (37.8%)
3	10 (27%)
4	7 (18.9%)
**Medication**	
- Betablockers	7 (18.9%)
- Calciumantagonists	7 (18.9%)
- Antihypertensive	18 (48.6%)
- Diuretics	4 (10.8%)
- Antiplatelet	23 (62.2%)
- NOAC/Vitamin K antagonists	0 (0%)
**LVEF**	
- Normal (>52%)	22 (59.5%)
- Mild dysfunction (40-52%)	6 (16.2%)
- Moderate dysfunction (30-40%)	1 (2.7%)
- Severe dysfunction (< 30%)	1 (2.7%)
- Unknown	7 (18.9%)
**History of**	
Peripheral vascular disease	2 (5.4%)
Hypertension	19 (51.4%)
Coronary artery disease	4 (10.8%)
Stroke	5 (13.5%)
Hyperlipidemia	14 (37.8%)
Diabetes	5 (13.5%)

BMI, Body Mass Index; NOAC, Novel oral anticoagulants; LVEF, Left Ventricular Ejection Fraction.

**Figure 1:**
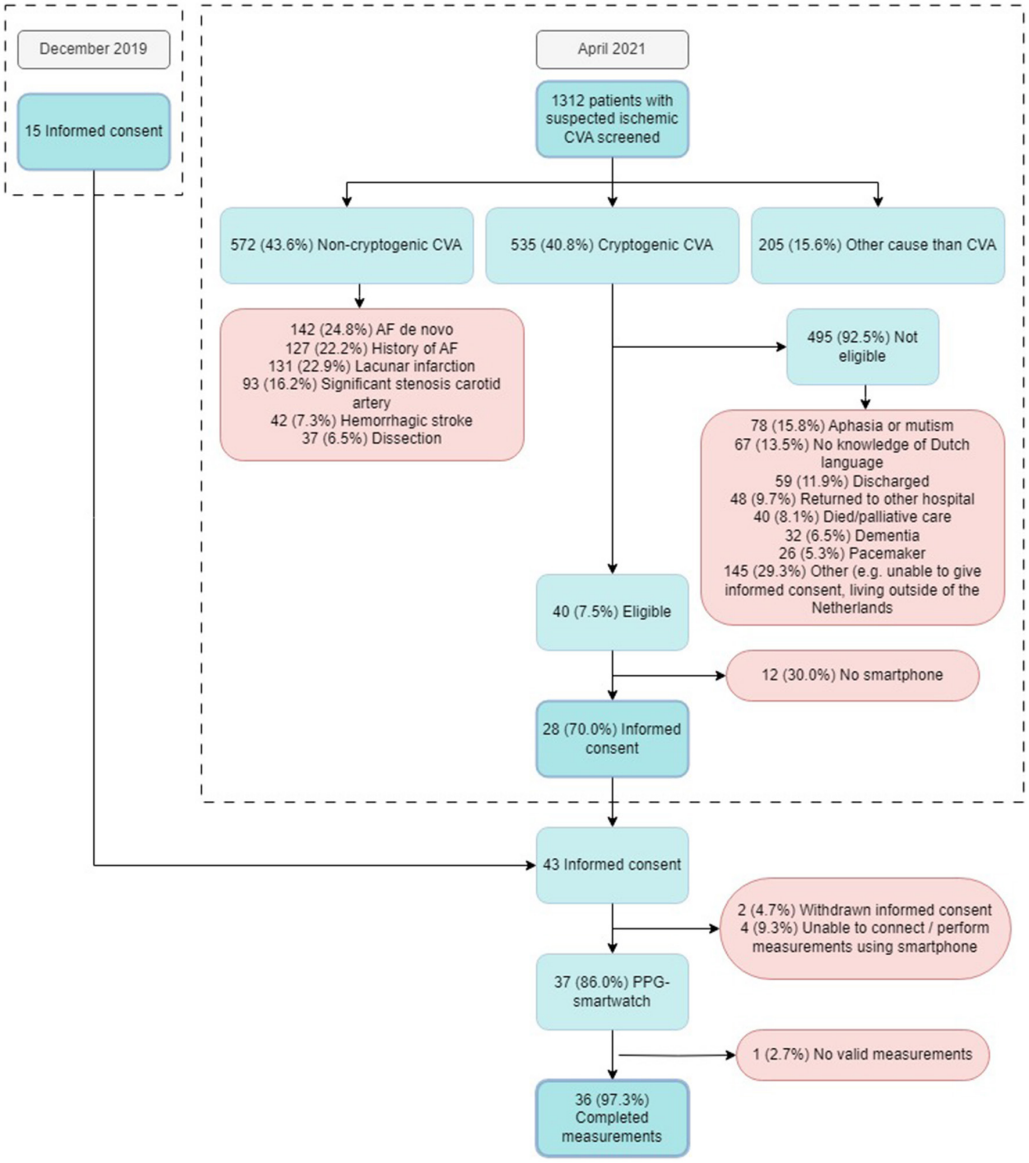
Flowchart of all patients screened and included in the study. CVA, Cerebral Vascular Accident; AF, Atrial Fibrillation; PPG, Photoplethysmography.

The detailed screening log documented 1,312 patients admitted to the neurology department with a primary diagnosis of cerebrovascular accident (CVA) or transient ischemic attack (TIA), of whom 572 (43.6%) had non-cryptogenic stroke. Among these, *de novo* AF was detected during hospital admission in 142 (24.8%), 127 (22.2%) had a history of AF, and 131 (22.9%) had a lacunar infarction. Two hundred and five patients (15.6%) were ultimately not diagnosed with a CVA. The remaining 535 patients (40.8%) were diagnosed with cryptogenic stroke, of whom 495 (92.5%) were deemed ineligible for study participation due to disabilities (e.g., aphasia, dementia) or logistical challenges (e.g., non-proficiency in Dutch, living outside of the Netherlands, no smartphone). Of the initially screened 1,312 patients, 28 (2.1%) were included in the study. A detailed analysis of inclusion and non-eligibility for study participation is shown in Figure 1.

Patient inclusion was prematurely ended on January 18, 2024 due to slower-than-expected inclusion rate. This report presents the initial results of the study.

### AF detection

**Figure 2:**
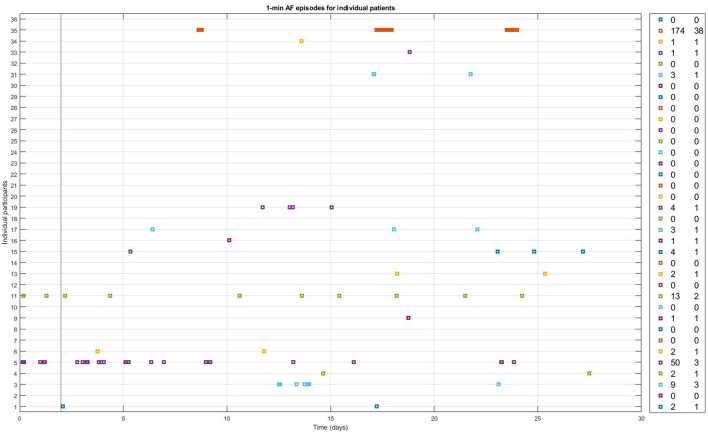
One-min episodes classified as AF by the FibriCheck AF algorithm v1.5.2. during the full 28 days of monitoring for each individual study participant with available PPG-measurements. Number of PPG-AF episodes and the maximum number of consecutive episodes of PPG-AF per patient are shown in the panel on the right.

During the 28-days of PPG monitoring, the median number of measurements recorded per patient was 4,496 [IQR 2,683; 8,849], with a median of 157 [IQR 107; 309] measurements per day. The median percentage of measurements deemed to be of insufficient quality was 38% [IQR 33%; 49%]. PPG-based AF was detected in 16 participants (43%) (Figure 2). The median number of AF episodes detected by 28 days of PPG monitoring in patients with AF detected was 2.5 [1.5; 6.5], leading to a median AF burden of 0.048% [0.019; 0.086]. The maximum number of consecutive AF episodes in an individual patient was 38, with a median of 1 [1; 1.5] consecutive episodes.

During the first 48 h, the FibriCheck algorithm identified AF in 2 (5.4%) of the patients. Those two patients had 3 and 14 episodes of AF during the first 48 h, with a burden of 0.32% and 1.46% respectively. In contrast, in none of the 36 patients (0%) AF was found during Holter monitoring during these 48 h.

### FibriCheck algorithm development

**Figure 3:**
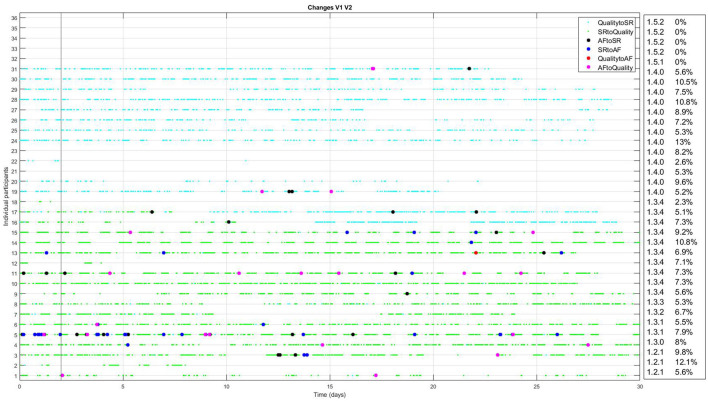
Changes between initial analysis and analysis using FibriCheck V1.5.2 for every individual participant with available PPG-data. The light blue dots represent reclassifications from insufficient quality to sinus rhythm (SR). Green dots represent reclassifications from SR to insufficient quality. Black dots represent reclassification from atrial fibrillation (AF) to SR and blue dots the reclassification from SR to AF. Red dots represent reclassification from insufficient quality to AF and pink dots the reclassification from AF to insufficient quality. In the right hand panel, the version of the FibriCheck algorithm which was available for the initial analysis is shown, followed by the percentage of PPG-measurements that were reclassified.

The FibriCheck algorithm was further developed during the study period, and initially data of each patient were analyzed using the most recent version of the FibriCheck AF algorithm at the time of inclusion. As a result, 3 patients were analyzed using v1.2.1, 1 using v1.3.0, 2 using v1.3.1, 1 using v1.3.2, 1 using v1.3.3, 10 using v.1.3.4, 13 using v1.4.0, 1 using v 1.5.2, and 4 using v1.5.2. After study closure, all patient data were re-analyzed using version 1.5.2. Changes between initial and final analysis are presented in Figure 3. Several episodes initially classified as AF were later reclassified to no-AF, and vice versa. Specifically, one episode was reclassified from AF to SR, and 26 episodes were reclassified from SR to AF (Table 2).

**Table 2:** The number of episodes (# eps) and that are reclassified between initial analysis and analysis using V1.5.2 and in which number of patients (# pat) these episodes were reclassified.

		**V1.5.2**					
		**SR**		**AF**		**Poor quality**	
		**# eps**	**# pat**	**# eps**	**# pat**	**# eps**	**# pat**
Initial	SR			26	9	9,170	18
algorithm	AF	1	1			40	8
	Poor quality	4,931	30	42	11		

SR, Sinus Rhythm; AF, Atrial Fibrillation; Poor quality, Insufficient Quality.

## Discussion

In this study, we aimed to assess the AF detection rate using a PPG-smartwatch compared to standard Holter monitoring in patients after cryptogenic stroke. The inclusion was stopped prematurely, due to a lower-than-expected inclusion rate. PPG-based AF was detected in 16 patients (43.2%) in 28 days of monitoring. However, the discrepancies found between 48 h Holter monitoring and concurrent PPG-based AF detection raised major concerns about the accuracy of PPG-based AF detection found in this study.

### Patient population

The study encountered challenges in patient inclusion, as only 7.5% of the screened patients with cryptogenic stroke were eligible to participate. This low inclusion rate suggests that the current population is not well-suited for screening using a PPG-smartwatch, although some non-eligibilities were inherent to the conduct of medical research and the inability to provide informed consent (e.g., aphasia, non-proficiency in Dutch), rather than true ineligibility for the use of this technology (e.g., access to a smartphone, dementia). If PPG-based AF detection using a smartwatch were ever to become standard care, informed consent for study participation would not be required and a significantly larger proportion of patients might be suitable for a PPG-based diagnostic strategy. Additionally, the inclusion of patients able to give informed consent inherently biases the study population toward those with milder strokes and fewer disabilities.

### PPG-AF

**Figure 4:**
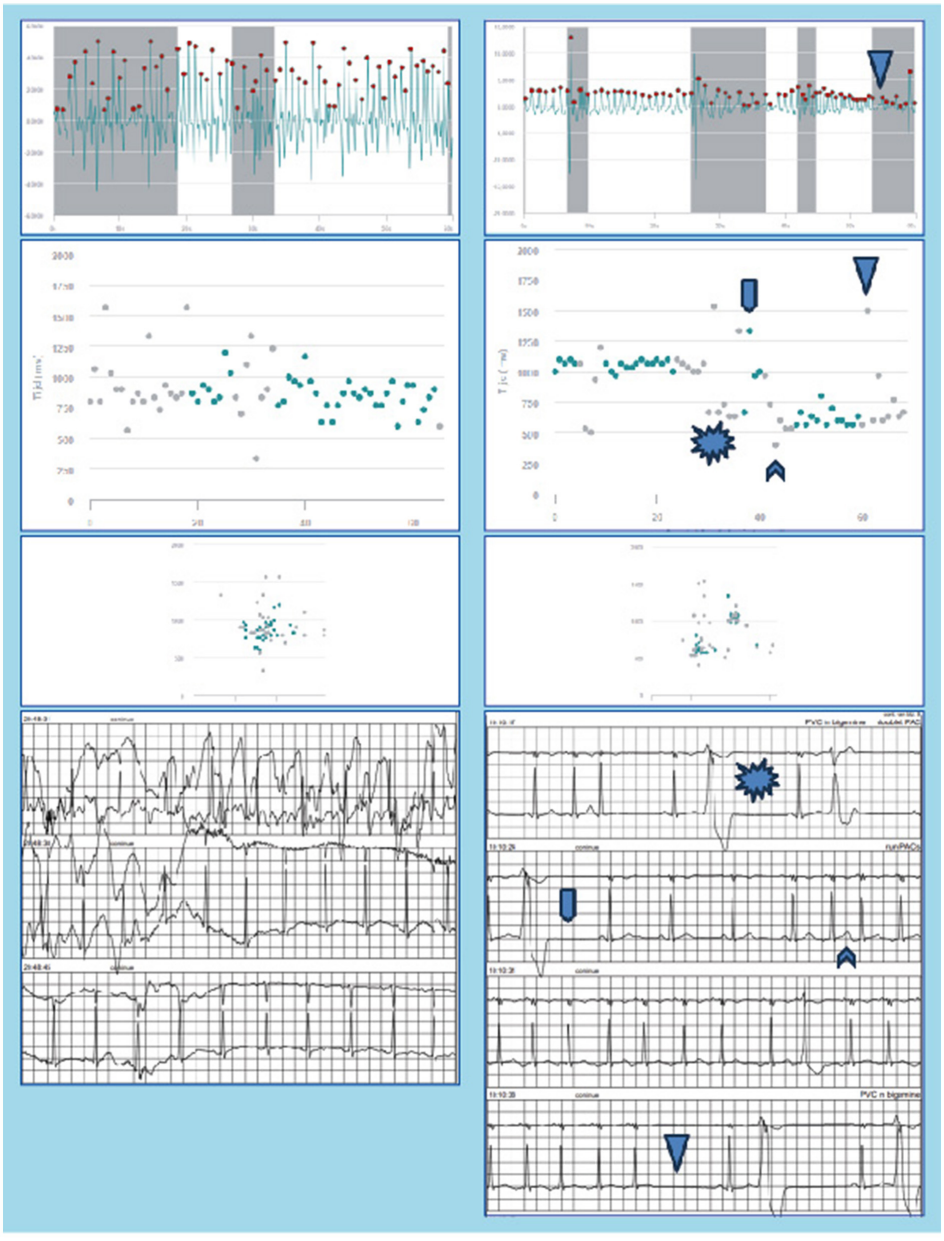
Raw PPG-signals, RR-intervals, Poincaré plot, and concurrent Holter recordings as measured during the first 48 h of monitoring are shown for two patients. The PPG signals were erroneously classified as AF by the FibriCheck algorithm. In the left hand panels the Holter shows noise, whereas in the right hand panels PAC/PAT and PVC can be observed.

In the 28 days of PPG monitoring using a smartwatch, PPG-based AF was detected in 16 (43%) of the participants, which is substantially higher than the previously reported 12.1% by Flint et al. (2012) or 14.4% by Gladstone et al. (2014) using prolonged monitoring after cryptogenic stroke. A possible explanation for this findings is that either noise from using the smartwatch during daily activities, or misinterpretation of premature atrial contractions (PACs) or premature ventricular contractions (PVCs), resulted in erroneous classification as AF by the PPG-algorithm. This hypothesis is substantiated by the fact that a discrepancy is observed between AF detection using PPG-based screening and Holter monitoring during the same 48 h period. PPG-based screening identified episodes of AF in the first 48 h that Holter monitoring did not identify, thus raising concerns about the accuracy and reliability of PPG-based AF detection using a smartwatch. Upon revisiting the Holter data, no false-negative AF was detected, and the episodes of PPG-based AF were found to correspond with noise or PACs and PVCs on the Holter recordings. Figure 4 shows two examples of PPG signals and their corresponding RR intervals interpreted as AF during the first 48 h of monitoring, and the simultaneously recorded Holter signals displaying noise (left hand panels) and PVCs (right hand panels).

Moreover, the percentage of measurements discarded due to insufficient quality by the FibriCheck algorithm was 38%. This exceeds the previously reported 5%−24% (Selder et al., 2020, 2023). This may result from increased motion artifacts and poor sensor contact during smartwatch use in daily activities, as the 5%−24% was found in controlled settings during shorter monitoring periods (Pereira et al., 2020). This notion is consistent with prior studies, such as Chang et al. (2022), who reported that nearly a quarter of PPG-smartwatch measurements during daily activities were discarded due to low signal quality, and that false positives were largely attributed to PACs and PVCs. A study by Zhao et al. (2024) showed significant differences in insufficient quality PPG-smartwatch measurements during day and night, with 43.0% valid measurements during the day and 76.1% during the night. Improvements in noise detection of smartwatch-PPG algorithms and stricter criteria for AF classification (e.g., requiring multiple consecutive measurements) might help reduce these discrepancies, but further validation is needed to establish clinically relevant cutoffs for the duration of episodes. The many versions of the FibriCheck algorithm during the study period and the consequent reclassification of several AF and SR episodes show that this is an ongoing process. In particular, the reclassifications from SR to insufficient quality (mainly v1.3.4) or insufficient quality to SR (mainly v1.4.0) show that noise detection is an important part of further algorithm development (Figure 3).

Given the questionable validity of AF detection using PPG-smartwatch monitoring, in addition to the ongoing discussions regarding anticoagulation in the prevention of stroke and balancing bleeding risk in patients with atrial high-rate episodes and AF detected during screening, it is not feasible to associate our PPG-based findings with therapeutic actions (Kirchhof et al., 2023; Healey et al., 2024; Svennberg et al., 2021; Svendsen et al., 2021).

### Limitations

The primary limitation of this study is the premature ending of inclusion. Given the small number of subjects, the results may be susceptible to bias. A larger sample size is needed to draw more robust and generalizable conclusions. The exclusion of patients unable to use a smartphone or smartwatch, as well as those with severe stroke-related disabilities, likely skewed the study population toward younger, relatively healthy and more technologically adept individuals. Furthermore, the high percentage of poor-quality PPG-smartwatch recordings during daily activities and the evolving nature of the FibriCheck algorithm complicate the interpretation of our findings.

## Conclusion

This study demonstrates a high rate (43%) of PPG-based AF detection during 28 days of monitoring in patients after cryptogenic stroke. However, the discrepancy in AF detection within the first 48 h of PPG monitoring, as compared to simultaneous Holter monitoring, raises important concerns regarding the validity of these PPG-identified AF episodes, suggesting they may represent misclassified noise or PACs/PVCs rather than true AF. Further refinement of the algorithm in noise detection and validation during daily activities are therefore needed before prolonged PPG-based AF detection using a smartwatch can reliably be used in clinical practice after cryptogenic stroke. In addition, the feasibility of this screening strategy is uncertain due to severe logistical and technological challenges, partly inherent to this patient population with stroke related disabilities, hindering its current application in clinical practice.

## Data Availability

The raw data supporting the conclusions of this article will be made available by the authors, without undue reservation.
