# Supplementary material for: Photoplethysmography-based atrial fibrillation detection in patients after crytpogenic stroke
Source: Front Stroke. 2024 Dec 5;3:1496003. doi: 10.3389/fstro.2024.1496003 (PMC12802793; doi:10.3389/fstro.2024.1496003)
Supplement: Supplementary file 1 [file Table_1.docx]

**SUPPLEMENTS**

|  | AF to SR | SR to AF | Quality to AF | AF to Quality |
| --- | --- | --- | --- | --- |
| 1 |  | 0.07% |  |  |
| 2 |  |  |  |  |
| 3 |  | 0.01% | 0.04% | 0.03% |
| 4 |  | 0.02% |  | 0.01% |
| 5 |  | 0.05% | 0.17% | 0.19% |
| 6 |  | 0.01% | 0.01% | 0.01% |
| 7 |  |  |  |  |
| 8 |  |  |  |  |
| 9 |  |  | 0.01% |  |
| 10 |  |  |  |  |
| 11 |  | 0.07% | 0.04% | 0.01% |
| 12 |  |  |  |  |
| 13 | 0.01% |  | 0.01% | 0.04% |
| 14 |  |  |  | 0.01% |
| 15 |  | 0.02% | 0.01% | 0.05% |
| 16 |  |  | 0.01% |  |
| 17 |  |  | 0.03% |  |
| 18 |  |  |  |  |
| 19 |  | 0.04% | 0.04% |  |
| 20 |  |  |  |  |
| 21 |  |  |  |  |
| 22 |  |  |  |  |
| 23 |  |  |  |  |
| 24 |  |  |  |  |
| 25 |  |  |  |  |
| 26 |  |  |  |  |
| 27 |  |  |  |  |
| 28 |  |  |  |  |
| 29 |  |  |  |  |
| 30 |  |  |  |  |
| 31 |  | 0.04% | 0.02% |  |
| 32 |  |  |  |  |
| 33 |  |  |  |  |
| 34 |  |  |  |  |
| 35 |  |  |  |  |
| 36 |  |  |  |  |

Table S1: the percentage of the total number of episodes per patient that are reclassified. AF to SR = Atrial Fibrillation to Sinus Rhythm, SR to AF = Sinus Rhythm to Atrial Fibrillation, Qualtiy to AF = Insufficient Quality to Atrial Fibrillation, AF to Quality = AF to insufficient Quality.
